# Enhancing Teamwork and Patient Safety Through TeamSTEPPS^®^: A Scoping Review of Benefits in Academic and Clinical Settings

**DOI:** 10.3390/nursrep16030079

**Published:** 2026-02-24

**Authors:** Leonor Velez, Patrícia Costa, Nuno Santos, Mafalda Inácio, Ana Rita Figueiredo, Susana Ribeiro, Paulo Cruchinho, Elisabete Nunes, Pedro Lucas

**Affiliations:** 1 Nursing Research Innovation and Development Centre of Lisbon (CIDNUR), School of Nursing, Universidade de Lisboa, 1990-096 Lisbon, Portugal; leonorvelez@campus.esel.pt (L.V.); patriciacosta@esel.pt (P.C.); n.santos@campus.esel.pt (N.S.); susanasantos@esel.pt (S.R.); enunes@esel.pt (E.N.); prlucas@esel.pt (P.L.); 2 Nursing Administration Department, School of Nursing, Universidade de Lisboa, 1600-190 Lisbon, Portugal; 3 Department of General Surgery and Gastroenterology, Instituto Português de Oncologia de Lisboa Francisco Gentil, 1099-023 Lisbon, Portugal; 4 Department of Internal Medicine, Hospital da Luz de Lisboa, Avenida Lusíada 100, 1500-650 Lisbon, Portugal

**Keywords:** TeamSTEPPS^®^, health care delivery, education, professional, interprofessional education, patient safety, review

## Abstract

**Background:** Teamwork promotes the quality and safety of care. The TeamSTEPPS^®^ program enhances communication and teamwork among healthcare professionals and students, as well as the associated benefits. Currently, there are no studies that comprehensively explore the benefits achieved through the implementation of TeamSTEPPS^®^ across different contexts (educational and clinical practice). **Objective**: This scoping review aimed to map the existing evidence on the benefits of implementing TeamSTEPPS^®^ in educational and professional settings, emphasizing its contribution to sustainable teamwork, patient safety, and organizational learning. **Methods**: A scoping review was conducted following the Joanna Briggs Institute methodology and reported according to the PRISMA-ScR guidelines. Searches were performed in CINAHL Ultimate, Medline Ultimate, Scopus, the Portuguese Open Access Scientific Repository, Web of Science and Psychology and Behavioral Sciences Collection, with no time restrictions. Studies were selected based on the PCC framework, focusing on healthcare students and professionals (Population), TeamSTEPPS^®^ implementation (Concept), and academic or clinical settings (Context). A descriptive and thematic analysis was used, enabling the identification of emerging categories and recurring patterns among the included studies. **Results**: Twenty-eight articles published between 2009 and 2025, predominantly from the United States of America and conducted in hospital settings, were found. The included studies comprised quantitative (n = 11), qualitative (n = 4) and quasi-experimental study (n = 13) designs. From the analysis, four thematic categories emerged: academic education, interprofessional education and simulation; professional transition and professional development; clinical implementation of the TeamSTEPPS^®^ program in real-world settings; and patient safety culture as a central focus. **Conclusions**: The available evidence suggests that the TeamSTEPPS^®^ program may strengthen teamwork and promote safe and high-quality care in both educational and clinical settings. While short-term training leads to immediate improvements in team dynamics, continuous training demonstrates greater long-term effectiveness. The consolidation of the TeamSTEPPS^®^ methodology relies on organizational commitment, leadership engagement, and the integration of interprofessional training from the academic level onward.

## 1. Introduction

The efficiency of healthcare delivery requires the implementation of effective teamwork. Working in teams enables the provision of coordinated, comprehensive, and safe healthcare [1], centered on the patient [2], which produces positive outcomes, for instance, among patients with symptoms associated with diabetes, chronic pain, and arterial hypertension [3]. However, inefficiency and inadequate communication can lead to misdiagnoses, increased numbers of falls, and incorrect patient identification [4]. The World Health Organization estimates that 3 to 16% of patients are affected by adverse events due to ineffective teamwork [5,6,7,8]. Thus, patient safety largely depends on the functioning of the work team. Campbell et al. [9] stated that 75% of deaths in hospital settings stem from inefficient teamwork and communication failures. Consequently, highly functional teams are less likely to commit medical errors [10], that is, unexpected events that may cause harm to the patient [11]. Conversely, dysfunctional teamwork promotes negative outcomes not only for patients but also for healthcare institutions [2].

In this context, a team is characterized as a group of individuals with common goals and complementary skills who assume responsibility for the benefits resulting from their joint work [12,13]. Teams differ in composition and are characterized by the duration of each member’s participation, structure, interpersonal interactions occurring during work, and functions defined by assigned tasks and established goals [14]. The concept of teamwork developed in response to the increasing complexity of care [15]. It is defined as a group of two or more individuals working toward a common goal [16,17]. It involves communication, trust, bonding, appreciation and respect for peers’ work, as well as collaboration among team members [18]. Teamwork and effective communication positively influence interprofessional collaboration [19].

Training is a strategy that develops effective teamwork skills, attitudes, and professional perceptions [10], ensuring the ability to analyze and evaluate the care provided to patients safely and with quality. Therefore, it must be developed from the academic stage [20,21,22,23].

To improve teamwork competencies, healthcare institutions seek training programs focused on group dynamics, as teamwork is considered a key component in reducing communication failures and enhancing safety [10,24]. According to the Organisation for Economic Co-operation and Development (OECD) [25], team training should include behavioral and knowledge-based competencies. Thus, to develop communication, collaboration, and teamwork skills, individual training should focus on teamwork competencies such as leadership, coordination, communication, collaboration, role definition, situation monitoring, and the use of effective communication [1,21]. Regarding team knowledge competencies, these include principles and concepts that enable members to understand each other’s roles, responsibilities, and limitations, share a common language, and develop effective coordination and collective decision-making strategies, enhancing the team’s overall performance [26,27,28]. These competencies involve understanding the functions performed within the team, the defined objectives, and the most appropriate ways for team members to collaborate [26,29].

To improve teamwork effectiveness and strengthen patient safety, in 2006, the TeamSTEPPS^®^ (Team Strategies and Tools to Enhance Performance and Patient Safety) training program was developed by the Agency for Healthcare Research and Quality, in collaboration with the U.S. Department of Defense [30,31]. This evidence-based program aims to enhance teamwork and communication skills among physicians and nurses [10], as well as students, thereby improving teamwork, communication, and consequently generating benefits for patient health [32]. It emphasizes that patient safety results from the relationship between effective communication and teamwork [33].

TeamSTEPPS^®^ interventions focus on team structure, communication, leadership, situation monitoring, and mutual support [30,34], incorporating innovative communication methods such as videoconferencing and active learning through simulation, discussion, and experience sharing [35].

Although it presents an innovative approach focused on team effectiveness, the implementation of TeamSTEPPS^®^ entails both challenges and opportunities. Regarding challenges, stress, distraction, fatigue [36], lack of trainee engagement [37], resistance to change, and limited financial or human resources [38] hinder the implementation and success of training.

Evidence suggests that the effectiveness of TeamSTEPPS^®^ implementation is influenced by active trainee engagement through shared lived experiences, curricular adaptability to diverse educational contexts, and its applicability to interprofessional communication training among healthcare professionals [31,36,39]. Program implementation and outcomes are commonly evaluated using standardized instruments such as the TeamSTEPPS@ Teamwork Attitude Questionnaire (T-TAQ) and the Team Performance Observation Tool (TPOT) [39,40], which assess five core teamwork dimensions: communication, leadership, mutual support, situation monitoring, and the team structure [30,31,39,41]. Although several reviews examining the TeamSTEPPS^®^ training program have been published between 2018 and 2023, no evidence syntheses prior to 2018 were identified, and existing reviews have not comprehensively mapped implementation strategies, evaluation instruments, and reported outcomes across healthcare contexts. Therefore, this scoping review aims to systematically map the existing evidence on the implementation, evaluation approaches, and outcomes of TeamSTEPPS^®^ training in healthcare settings.

In 2018, Wesch et al. [32] conducted a systematic literature review focusing on studies addressing interprofessional education using TeamSTEPPS^®^ and healthcare simulation. In 2019 and 2020, two integrative literature reviews were conducted: Parker et al. [37], who described the influence of TeamSTEPPS^®^ on clinical error rates and patient satisfaction, and Ross et al. [20], who explored outcomes resulting from training future healthcare professionals. Also in 2019, a scoping review [39] was conducted with the aim of assessing the implementation and evaluation of the TeamSTEPPS^®^ training program in interprofessional education. From 2020 to 2023, three additional literature reviews were published: Brooks et al. [10], analyzing the effectiveness of TeamSTEPPS^®^ in improving perceptions of teamwork; Kriz [42], investigating the effectiveness of TeamSTEPPS^®^ simulations in improving nurses’ teamwork attitudes and performance during cardiorespiratory emergencies; and Yuanasika & Rosa [19], highlighting the program’s influence on healthcare teams regarding perceptions and service performance.

Unlike previous reviews published between 2018 and 2023, which primarily focused on specific educational contexts, simulation-based interventions, or short-term outcomes of TeamSTEPPS^®^ training, this scoping review adopts an integrated perspective across academic and clinical settings. By mapping reported benefits along the educational-to-practice continuum, this review not only confirms well-documented outcomes such as improvements in communication and teamwork perceptions but also synthesizes evidence related to sustainability, leadership involvement, and organizational embedding of TeamSTEPPS^®^ practices.

A search for completed or ongoing reviews was conducted across four review registry databases—PROSPERO, OSF Registries, INPLASY, and the Cochrane Database of Systematic Reviews—to identify published scientific evidence on the benefits of the TeamSTEPPS^®^ program in academic and professional settings. Considering that undergraduate training generally occurs within nursing teams, the absence of a comprehensive perspective linking initial education and professional practice reveals a knowledge gap essential to understanding the program’s effects across the formative and care continuum, thereby justifying the relevance and innovation of this scoping review.

The published evidence on TeamSTEPPS^®^ in recent years has addressed various contexts, educational levels, and methodological designs. This heterogeneity hinders understanding of: (1) the benefits reported; (2) which dimensions of teamwork were effectively improved; (3) which methods were used to evaluate the benefits promoted by TeamSTEPPS^®^; and (4) in which contexts TeamSTEPPS^®^ implementation yields greater effects. Furthermore, no evidence articulates the benefits of TeamSTEPPS^®^ across the continuum from undergraduate training to professional practice, representing a relevant gap for both healthcare practice and research.

In this sense, a Scoping Review is the most appropriate method to map the extent, type, distribution, and nature of existing evidence without limiting the inclusion of studies to a single methodological design. This approach enables the identification of convergences, divergences, and gaps in the literature, clarifies concepts, supports decision-making in educational and clinical contexts, and guides future interventions and scientific production. Therefore, conducting this review is both relevant and necessary to consolidate the existing knowledge on TeamSTEPPS^®^ and to strengthen the development of safe, effective, and collaborative healthcare teams throughout the entire educational and clinical continuum.

Accordingly, a scoping review was conducted to compile existing scientific evidence, identify gaps in the literature, and generate knowledge to foster innovation in practice and the design of healthcare interventions. This methodology was deemed the most appropriate as it allows for the mapping of broad and heterogeneous evidence in complex fields, facilitating the clarification of key concepts and the identification of research gaps, consistent with the JBI methodological framework [43].

To this end, the following review question was formulated: What is the available evidence on the benefits of implementing TeamSTEPPS^®^ in educational and professional contexts, and how have these benefits been described, measured, and reported in the literature? The objective is to acquire knowledge that facilitates the selection and implementation of TeamSTEPPS^®^ to improve health outcomes and develop teams capable of effective collaboration from undergraduate training to professional practice.

## 2. Materials and Methods

The scoping review followed the methodology proposed by the Joanna Briggs Institute (JBI) for reviews of this nature [43] and was reported in accordance with the Preferred Reporting Items for Systematic Reviews and Meta-Analyses Extension for Scoping Reviews (PRISMA-ScR) [44]. A completed PRISMA-ScR checklist is provided to document adherence to these reporting standards [see Supplementary Files]. The review title was registered on the Open Science Framework platform (https://doi.org/10.17605/OSF.IO/XTJUR) (accessed on 1 February 2026). The methodological framework proposed by Arksey and O’Malley [45] and refined by the JBI [46] was adopted for the descriptive and thematic analysis of the data addressing the review question.

The review question was developed using the PCC framework, in which “P” (Population) refers to healthcare students and professionals; “C” (Concept) corresponds to the implementation of the TeamSTEPPS^®^ training program; and “C” (Context) refers to academic education and clinical practice.

This review aims to map scientific evidence on the outcomes produced by the TeamSTEPPS^®^ training program in educational and professional contexts.

### 2.1. Eligibility Criteria

The eligibility criteria were defined based on the PCC framework, as proposed by the Joanna Briggs Institute (JBI):

- Participants: Articles focusing on healthcare students (undergraduate, master’s, and postgraduate courses) and/or healthcare professionals were included. Studies whose target population did not consist of healthcare students or professionals were excluded.
- Concept: Studies whose primary objective was to assess the outcomes of implementing the TeamSTEPPS^®^ program and that reported at least one measurable outcome were considered eligible. Studies that did not specifically mention TeamSTEPPS^®^ were excluded.
- Context: Studies conducted in educational contexts (academic training) and/or clinical settings (professional healthcare practice) were included. Studies in which TeamSTEPPS^®^ was implemented outside the healthcare sector were excluded.

### 2.2. Types of Sources

This scoping review included empirical studies with different methodological designs, namely quantitative, qualitative, quasi-experimental studies, case studies, practice improvement projects, developmental studies, cross-sectional studies, time series analyses, texts and opinion articles, master’s and doctoral theses and dissertations, unpublished studies, books, and book chapters.

### 2.3. Search Strategy

The search strategy aimed to identify articles reporting outcomes resulting from the implementation of the TeamSTEPPS^®^ program. The search was conducted in three distinct stages.

First, the CINAHL Ultimate (EBSCO) and Medline Ultimate (EBSCO) databases were used to analyze keywords appearing in titles and abstracts. The terms identified in titles and abstracts enabled the development of a comprehensive search strategy applicable across the three databases. The research strategy used is described in Table 1.

Subsequently, a broader search was conducted using the previously identified keywords in the following databases: CINAHL Ultimate (EBSCO), Medline Ultimate (EBSCO), Scopus, the Portuguese Open Access Scientific Repository (RCAAP), Web of Science and Psychology and Behavioral Sciences Collection. The inclusion of RCAAP was a deliberate strategy to access grey literature, including national theses and dissertations, ensuring a broader coverage of TeamSTEPPS^®^ implementations that may not yet be published in international peer-reviewed journals. The search was performed on 23 December 2025. For each database, a specific search string was developed (Table 1).

The search was limited to articles providing full-text access, available bibliographic references, and open-access availability. No time restrictions were applied, as the objective of this scoping review was to comprehensively map all existing literature on the topic under study [47]. Studies published in English, Portuguese, and Spanish were considered eligible. These languages were selected based on the review team’s linguistic expertise to maintain high standards of accuracy during data extraction and interpretation.

No temporal or geographical restrictions were applied to ensure an exhaustive mapping of the existing literature on the topic under study.

### 2.4. Bibliographic Source Selection

The search strategy yielded a total of 675 articles. Their references and citations were exported from the respective databases and imported into the online platform Rayyan^®^ (version 1.6.3). Using the Rayyan^®^ platform, duplicate records were removed. The remaining articles were screened according to the previously defined eligibility criteria. This process was carried out independently by two reviewers (L.V., P.C.).

After duplicate removal, 115 articles were identified. Subsequently, based on the analysis of titles and abstracts, 54 articles were selected for full-text review. Of these, 23 articles met the inclusion criteria.

In adherence to the JBI scoping review framework, we chose not to perform a formal quality appraisal of the literature. While we recognize that this limits our insight into the internal validity of individual studies, our priority was to map the full breadth of the evidence and capture the diverse scientific evidence.

### 2.5. Data Extraction

Data were extracted by two independent reviewers using a data extraction table based on the JBI model [48], developed in Microsoft Word^®^. Disagreements were resolved through consensus or, when necessary, by consultation with a third reviewer. The table was structured according to bibliographic details, methodological characteristics, contextual information, research instruments, key concept(s) and reported outcomes [see Supplementary Files].

## 3. Results

**Figure 1:**
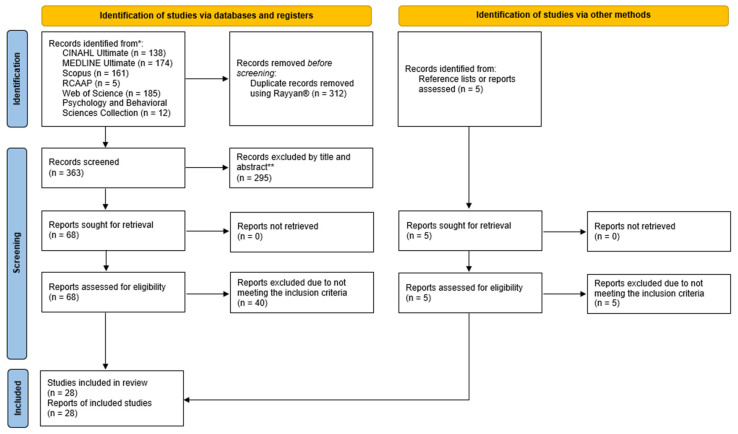
PRISMA 2020 flow diagram for new systematic reviews, which included searches of databases, registers, and other sources [49]. The asterisk indicates the databases included. * databases included. ** Reports excluded after title and abstract screening.

The PRISMA Flow Diagram (Figure 1) illustrates the process of identification, screening, eligibility, and inclusion of studies, in accordance with Page et al. [49].

The articles included in this scoping review were published between 2009 and 2025 and originated from various countries, predominantly from the United States (USA) (67.9%; n = 19) [50,51,52,53,54,55,56,57,58,59,60,61,62,63,64,65,66,67,68], Egypt (3.6%; n = 1) [69], Thailand (3.6%; n = 1) [70], Norway (10.7%; n = 4) [71,72,73,74], Australia (3.6%; n = 1) [75], Brazil (3.6%; n = 1) [76] and India (3.6%, n = 1) [77].

Most of the articles originated from countries outside the European continent (85.7%; n = 24), with 19 of them conducted in the USA [50,51,52,53,54,55,56,57,58,59,60,61,62,63,64,65,66,67,68] one in South America [76], one in Oceania [75], two in Asia [70,77] and one in Africa [69]. Only 14.3% of the studies originated from Europe, specifically from Norway [71,72,73,74].

Of the total number of articles, 14 (50%) [50,56,57,58,60,61,63,64,69,70,71,73,76,77] were published within the last five years, indicating strong and current scientific relevance.

This scoping review comprises studies with the following designs: quantitative (39.3%; n = 11) [50,53,55,60,61,62,63,64,65,68,73], qualitative (14.3%; n = 4 [54,66,74,75], and quasi-experimental studies (46.4%; n = 13) [51,52,56,57,58,59,67,69,70,71,72,76,77].

Regarding participants, 14 (50%) of the included studies reported included healthcare professionals from multidisciplinary teams (e.g., physicians, nurses, pharmacists, healthcare assistants, and respiratory therapists). However, since not all articles reported the number of participants, it was not possible to accurately estimate the total global sample size. Among the studies that provided this information, there was a predominance of practicing healthcare professionals, representing almost the entirety of the reported participants (over 30,000 across the included studies).

Also, 14 (50%) of the included studies reported students from medicine, nursing, and health management programs, as well as research conducted in specific units such as assisted reproduction centers.

**Figure 2:**
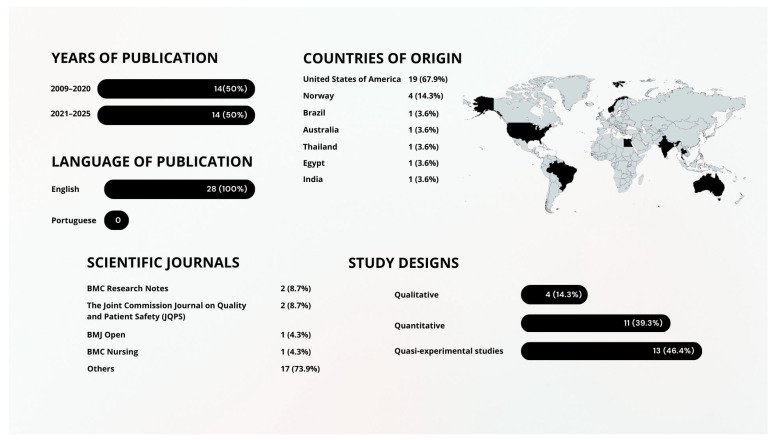
Summary of Data on the Included Articles.

Figure 2 summarizes the characterization of the studies included in this scoping review.

Regarding the context, twelve studies (42.9%) were conducted in academic settings [50,51,52,54,56,57,63,66,68,70,71,76], during a professional internship [69,77], thirteen (46.4%) in hospital environments [53,59,60,61,62,64,65,67,69,72,73,74,75] and one (3.6%) in ambulatory assisted reproduction centers [55] [see Supplementary Files].

Based on the analysis of the 28 articles included in this review, four thematic categories emerged:

- Academic education, interprofessional education, and simulation;
- Professional transition and professional development;
- Clinical implementation of TeamSTEPPS^®^ in real-world settings;
- Patient safety culture as a central focus.

The categorization was performed through thematic content analysis, following the principles proposed by Braun and Clarke [78]. This process involved the systematic reading of the extracted data, followed by the identification of meaning units related to the study objectives. These units were then coded, grouped into subthemes, and finally integrated into the four overarching categories that emerged from the synthesis.

This analytical procedure, both intuitive and deductive in nature, enabled a coherent organization of the evidence and ensured the representativeness of the different educational and clinical contexts in which the TeamSTEPPS^®^ program was implemented.

Table 2 presents a summary of the contributions of the 28 included studies, organized according to the identified thematic categories.

## 4. Discussion

This scoping review aimed to map scientific evidence on the outcomes produced by the implementation of the TeamSTEPPS^®^ training program in both clinical practice and academic settings. Accordingly, 28 articles were included, and their analysis led to the identification of four key themes: (1) academic education, interprofessional education, and simulation; (2) professional transition and professional development; (3) clinical implementation of TeamSTEPPS^®^ in real-world settings; and (4) patient safety culture as a central focus.

These categories are considered to represent core dimensions for understanding the benefits derived from the implementation of the program. Consequently, this categorization serves as a fundamental framework for the development and implementation of innovative and effective training strategies tailored to the needs and realities experienced by healthcare students and professionals.

Across the four identified thematic categories, the analysis showed that TeamSTEPPS^®^ was implemented in both academic and clinical contexts, involving healthcare students and professionals from multiple disciplines, including physicians, nurses, pharmacists, and allied health professionals. In academic and simulation-based settings, the most relevant findings included improvements in teamwork attitudes, communication skills, leadership behaviors, and confidence in interprofessional collaboration. Studies focusing on professional transition and development highlighted immediate post-training gains in teamwork and patient safety perceptions, although several reported a decline over time without ongoing reinforcement.

In real-world clinical settings, TeamSTEPPS^®^ implementation was associated with enhanced interprofessional communication, leadership engagement, and the adoption of standardized teamwork practices, particularly when integrated at the organizational level. Across themes, patient safety culture emerged as a central outcome, with improvements in safety awareness, communication openness, and organizational learning, especially in studies emphasizing continuous training and leadership involvement.

This review both confirms and extends findings from previously published reviews and studies. In line with earlier reviews, improvements in communication, teamwork perceptions, and interprofessional collaboration were commonly reported following the implementation of TeamSTEPPS^®^. In addition to these well-documented benefits, the present synthesis brings attention to less extensively examined dimensions, particularly the role of leadership engagement, mandatory and recurrent training, and organizational integration in supporting the longer-term sustainability of reported outcomes.

Across the included studies, a consistent pattern emerged regarding the sustainability of reported outcomes. Improvements in teamwork attitudes, perceptions of communication, and confidence in teamwork behaviors appeared particularly sensitive to decline over time when training was not reinforced. In contrast, longer-term maintenance of reported benefits was more frequently described in studies that emphasized leadership engagement, mandatory and recurrent training, and the integration of TeamSTEPPS^®^ practices into organizational routines and safety processes.

### 4.1. Academic Education, Interprofessional Education, and Simulation

Academic education in the health sciences has evolved by incorporating innovative methodologies that foster the development of leadership, communication, and collaborative practice among professionals. These competencies are considered fundamental to achieving safety and quality in patient care. In this context, interprofessional education and simulation, both in academic and professional environments, as used in the TeamSTEPPS^®^ training program, have emerged as effective pedagogical strategies for achieving excellence in care delivery [50,52,53,54,56,57,58,63,66,68,70,76].

In the studies analyzed, both healthcare professionals (e.g., physicians, nurses, and pharmacists) [50,53,71,77] and students in medicine, nursing, physiotherapy, occupational therapy, and other health sciences [51,54,58,63,66,68,76] recognized simulation as a means of achieving significant improvements in teamwork performance and, consequently, in care outcomes [50,54,58,63,66,68,76].

Within this academic context, Brock et al. [52] described and demonstrated the effectiveness of interprofessional team training through simulation, providing evidence that structured TeamSTEPPS^®^-based simulation can positively influence teamwork-related outcomes in academic settings. In line with these findings, Volino et al. [50] reported that the application of TeamSTEPPS^®^ within an interprofessional simulation involving pharmacy and physician assistant students led to significant improvements in attitudes, perceived communication skills, and teamwork competencies.

Building on these findings, Karlsen et al. [71] conducted a longitudinal quasi-experimental study within a Bachelor of Nursing program, demonstrating that the systematic and curriculum-integrated implementation of TeamSTEPPS^®^ over a 24-month period led to statistically significant and sustained improvements in students’ attitudes toward teamwork. Positive effects were observed across core TeamSTEPPS^®^ domains, including team structure, leadership, and situation monitoring, reinforcing the importance of early and continuous exposure to structured teamwork training in academic settings. In a similar academic context, Mahmood et al. [77] evaluated an interprofessional simulation education module based on the TeamSTEPPS^®^ framework for medical and nursing undergraduates, reporting statistically significant improvements in teamwork performance, communication skills, and interprofessional competencies following the intervention.

According to Momim & Nguyen [63], medical and respiratory therapy students highlighted the repetition of teamwork training through the TeamSTEPPS^®^ program as essential for consolidating learning and standardizing interprofessional relationships. This training was based on clinical simulation and debriefing (reflection after simulations). Participants were exposed to a hypoxia case during the simulation. Similarly, Gonçalves et al. [76] stated that teamwork training through TeamSTEPPS^®^, combined with simulation, improved the performance of future healthcare professionals. After a lecture on the TeamSTEPPS^®^ program, each student acted as the leader in a simulated emergency (resuscitation). The authors reported improved team performance and a 34.2% reduction in response time during a simulated cardiac arrest. Conversely, the group of medical students who only received basic resuscitation training showed a 33.6% reduction in the total time for initial actions. These results reinforce that teamwork training is crucial for achieving better health outcomes and for enhancing team performance in care settings.

The use of virtual platforms to prepare health students for effective and safe care delivery also emerged as a relevant innovation for academic education and the future of healthcare. The study by Umoren et al. [66] demonstrates how interprofessional education can be enhanced through virtual simulation, using eight clinical cases. Prior to the simulation, students received training in team communication based on the TeamSTEPPS^®^ program. The findings showed that simulation enables the recognition of TeamSTEPPS^®^ communication tools, particularly the SBAR technique, strengthening students’ awareness of the importance of structured and effective communication. Moreover, it facilitated the dissemination of interprofessional teamwork knowledge. This was corroborated by Forstater et al. [57], Foltz-Ramos et al. [56] and Jitwiriyanont et al. [70], who expanded the evidence on the effectiveness and pedagogical versatility of TeamSTEPPS^®^-based simulations.

Forstater et al. [57] demonstrated that virtual interprofessional simulations produced outcomes equivalent to those of in-person sessions, showing improvements in patient safety knowledge, teamwork perception, and confidence in assertive communication within clinical contexts. Complementarily, Foltz-Ramos et al. [56] found that integrating role-play sessions prior to simulation increased students’ confidence in using TeamSTEPPS^®^ tools such as SBAR and promoted more positive attitudes toward interprofessional teamwork. Jitwiriyanont et al. [70] concluded that simulation activities based on the TeamSTEPPS^®^ program improved students’ tone of voice, enhancing courtesy, assertiveness, and interprofessional communication.

To support educators in implementing interprofessional activities, Davis et al. [54] described how three health universities incorporated the TeamSTEPPS^®^ program into their interprofessional education curricula, using diverse instructional formats—online, flipped classroom, and observed simulation practice. Results showed improved interprofessional performance, student satisfaction, and teamwork attitudes toward achieving shared goals. Similarly, Jernigan et al. [51] evaluated a large-scale, foundational interprofessional education program explicitly structured around the TeamSTEPPS^®^ framework, demonstrating significant improvements in students’ teamwork attitudes across all core TeamSTEPPS^®^ domains, as well as substantial acquisition of TeamSTEPPS^®^ knowledge and readiness for future collaborative practice.

At a private U.S. university, interprofessional education was integrated into the medical and nursing curricula throughout the academic trajectory. Williams et al. [68] highlighted team training as a means of addressing the deficit in role clarification among students. Participants underwent a one-week intensive TeamSTEPPS^®^ course focusing on the program’s core principles and engaging in simulation scenarios (hypertensive crisis or cardiac arrest). During each simulation, students practiced standardized communication using SBAR, mutual support, task definition and delegation, and final debriefing reflection. Williams et al. [68] reported that, following the intensive interprofessional education session, students were more willing to share knowledge and recognized collaboration as a key factor in patient safety. The training also enhanced students’ reflective abilities and helped faculty identify areas for improvement in interprofessional education.

Similarly, a study conducted in a pediatric unit evaluated the implementation of the TeamSTEPPS^®^ program among various professional categories. The focus was interprofessional training on four TeamSTEPPS^®^ competencies: leadership, situation monitoring, mutual support, and communication. The eight-week training included videos, simulated clinical cases, and debriefing sessions. Clapper et al. [53] concluded that the implementation of TeamSTEPPS^®^ improved professionals’ knowledge, communication, and peer support, thereby enhancing patient safety and reducing adverse events.

Fowler et al. [58] described another innovative experience in which academic and professional worlds intersected through a hospital quality improvement project. Approximately 137 students from nursing, medicine, pharmacy, and physiotherapy were trained in TeamSTEPPS^®^ and assessed teamwork within a hospital unit needing improved interprofessional collaboration. By observing and applying TeamSTEPPS^®^ competencies in professional contexts, students contributed to improved team performance regarding structure, communication, leadership, mutual support, and situation monitoring. These improvements persisted months after the intervention. This experience not only helped students understand the link between interprofessional learning and real-world care but also enabled them to recognize each member’s role and responsibilities. Furthermore, it provided nurse managers with valuable insights for improving collaboration, enhancing health outcomes, and fostering a positive clinical environment for both professionals and patients.

Overall, these findings reinforce that the structured integration of TeamSTEPPS^®^ into academic programs constitutes an effective strategy for developing collaborative competencies among students and professionals from diverse health disciplines.

### 4.2. Professional Transition and Professional Development

Hassan et al. [69] evaluated the impact of the TeamSTEPPS^®^ program on newly graduated nurses, that is, professionals in the transition phase from academia to clinical practice. Over a seven-week period, the newly graduated nurses participated in several pedagogical strategies, including lectures, discussions, and simulations. Each strategy focused on the four TeamSTEPPS^®^ core competencies: leadership, communication, situation monitoring, and mutual support. The study revealed significant improvements immediately after training, particularly in perceptions of teamwork and patient safety culture. These results were sustained for two months following the intervention. The findings also underscored the importance of nurse managers implementing the program systematically to ensure the integration of these competencies into the daily clinical practice of novice professionals. The authors concluded that TeamSTEPPS^®^ proved to be a valuable tool in facilitating the transition of newly graduated nurses into the workforce.

The study by Harvey et al. [59] analyzed the effect of advanced trauma nursing training, integrated with TeamSTEPPS^®^ education, on the performance of resuscitation teams in hospital settings. The intervention included didactic sessions, high-fidelity simulations, and interprofessional exercises with two types of scenarios. Nurses demonstrated greater confidence and improved performance in leadership and communication six months after the course; however, these gains decreased after twelve months, suggesting the need for periodic reinforcement. The results showed that integrating TeamSTEPPS^®^-based training promotes improved confidence and performance among trauma teams (nurses, physicians, pharmacists, and others). However, unlike the findings of Hassan et al. [69], team performance improvements declined after six and twelve months, highlighting the necessity for biannual reinforcement of teamwork dynamics based on TeamSTEPPS^®^ training in this environment.

Matzke et al. [61] developed a quality improvement project in an emergency and trauma center to assess team members’ perceptions of collaboration and communication after a one-hour educational session on TeamSTEPPS^®^. The training, adapted to the emergency care context, emphasized tools such as SBAR, leadership, situation monitoring, and mutual support. The study showed improvements in teamwork perceptions and collaboration; however, leadership, team structure, and mutual support did not show significant improvement post-training. Despite these less positive outcomes, the authors concluded that even brief training interventions can have a positive impact on professionals’ perceptions of teamwork and communication.

Together, these three studies highlight the relevance of TeamSTEPPS^®^ in professional development, as it fosters the growth of collaborative competencies. While Hassan et al. [69] emphasized the consolidation of a safety culture among newly graduated nurses, Harvey et al. [59] demonstrated the need for ongoing reinforcement to maintain health and team performance outcomes. Conversely, Matzke et al. [61] showed that even short-term interventions can yield meaningful improvements in teamwork perceptions. In summary, these studies reveal that the TeamSTEPPS^®^ training program supports professional development, ensuring quality and safety in collaborative healthcare practice.

### 4.3. Clinical Implementation of TeamSTEPPS^®^ in Real-World Settings

The study conducted by Snow et al. [64] described how the implementation of the TeamSTEPPS^®^ training program improved interprofessional communication within a perioperative setting. In this quality improvement project, videos depicting emergency situations were used to help healthcare professionals (physicians, nurses, and healthcare assistants) enhance their team communication. Participants reported greater confidence in using TeamSTEPPS^®^ tools, as well as the intention to apply them in future practice. Although the results focused on participants’ perceptions, the study demonstrated that virtual simulation is a viable strategy for fostering team training in operating room environments.

In a similar clinical context, Aaberg et al. [72] investigated the impact of an interprofessional teamwork intervention in a surgical unit through the implementation of TeamSTEPPS^®^. The training, delivered by unit leaders, included didactic sessions, high-fidelity simulations, and supporting materials related to the program’s content and tools. Six months after the intervention, changes were observed in the structure of interprofessional teamwork, including the establishment of daily interdisciplinary meetings. A follow-up training session was conducted eleven months later to reinforce the importance of the program. Aaberg et al. [72] concluded that phased and reinforced training enhances the effectiveness of teamwork.

In a subsequent study, Aaberg et al. [73] also implemented TeamSTEPPS^®^, with evaluations conducted at six and twelve months post-training. The objective was to assess physicians’, nurses’, and healthcare assistants’ perceptions of teamwork and patient safety culture. All participants attended a six-hour didactic session on TeamSTEPPS^®^ concepts, followed by simulations, debriefings, and clinical practice. After twelve months, improvements were noted in three teamwork dimensions: (1) situation monitoring, (2) mutual support, and (3) communication. However, no improvements were found in team decision-making. Regarding patient safety culture, significant improvements were observed in communication and continuous learning, along with a notable increase in managers’ engagement in patient safety actions. Interestingly, after the program’s implementation, there was an increase in reported adverse events, suggesting an enhanced reporting culture and, consequently, an improved patient safety culture.

Weaver et al. [67] examined the impact of TeamSTEPPS^®^ training on surgical teams. The four-hour intervention included role-playing and clinical simulations focused on the program’s core tools: communication, leadership, mutual support, and situation monitoring. Following training, both the quantity and quality of pre-surgical briefings improved, as did professionals’ perceptions of patient safety culture and interprofessional teamwork attitudes. The study found that 81% of participants reported higher confidence in teamwork after training, while 52% felt confident enough to teach TeamSTEPPS^®^ to others. As a result of these improvements, the intervention hospital made TeamSTEPPS^®^ training mandatory for all staff (clinical and non-clinical) to promote safe and effective care.

Similarly, Ballangrud et al. [74] described healthcare professionals’ experiences after TeamSTEPPS^®^ training in a hospital setting. Improvements were observed in leadership, resource management, communication, and the use of standardized tools such as ISBAR. Nurses reported greater independence in decision-making processes, although physicians, nurses, and healthcare assistants experienced difficulties applying ISBAR during emergency situations.

A study conducted in a mental health setting [75] implemented TeamSTEPPS^®^ through an intensive two-and-a-half-day training session for a multidisciplinary team. Each participant was then responsible for disseminating the knowledge among colleagues. The intervention resulted in the adoption of standardized communication using the SBAR technique, restructured multidisciplinary meetings, improved leadership, and reduced mechanical restraint rates. Despite these positive outcomes, the study noted limited adherence from hospital management, suggesting that sustained managerial engagement is essential for long-term success.

Although TeamSTEPPS^®^ is typically delivered in four to six hours, Mayer et al. [62] implemented a two-and-a-half-hour version in a pediatric intensive care unit. A “change team” of 17 members from three professional categories was responsible for cascading the training. After one month, team performance improved, and these gains persisted at six and twelve months. Additionally, improvements were noted in two quality indicators: reduced ECMO initiation time and lower hospital infection rates. However, the authors warned that working with untrained staff hindered the overall progress of team performance.

Kwon and Duzyj [60] demonstrated the benefits of implementing this innovative program in obstetric settings. Their adapted TeamSTEPPS^®^ training consisted of two 90-min sessions (totaling 180 min). Although participants expressed positive perceptions of teamwork, differences were observed among professional categories, with nurses showing a more critical perspective toward safety behaviors after training—possibly reflecting greater awareness of patient safety responsibilities. The study found no significant differences in postpartum hemorrhage rates, suggesting that clinical outcomes may be limited in the short term despite positive attitudinal changes.

In contrast to shorter interventions, Hassan et al. [69] implemented TeamSTEPPS^®^ at the organizational level over a 15-month period, integrating it as part of the institution’s annual mandatory training and orientation. The results revealed improvements in communication, feedback, and commitment to patient safety, confirming the crucial role of nursing and medical leadership in sustaining large-scale implementation.

Finally, Dodge et al. [55] evaluated the implementation of TeamSTEPPS^®^ in reproductive health centers over two years. The intervention involved establishing a change team, participating in informational webinars, and conducting SWOT analysis exercises. The findings reported increased patient identification pauses, use of standardized communication, improved teamwork perceptions, regular team meetings, and greater patient satisfaction. The long-term sustainability of these improvements highlighted the effectiveness of implementations supported by strong organizational engagement and continuous reinforcement.

In summary, these ten studies demonstrate that implementing the TeamSTEPPS^®^ training program in real clinical settings leads to improvements in communication, leadership, safety culture, and teamwork perceptions, despite variations in format, intensity, and duration. Short interventions generate immediate gains, whereas longer programs highlight the need for ongoing reinforcement to maintain training outcomes over time. The benefits of TeamSTEPPS^®^ have been documented across diverse contexts—from surgical and intensive care units to mental health and postpartum care—confirming its adaptability and impact on clinical performance and patient safety.

### 4.4. Patient Safety Culture as a Central Focus

The study by Hassan et al. [69] evaluated the impact of implementing TeamSTEPPS^®^ among newly graduated nurses, focusing specifically on the culture of patient safety. The results demonstrated immediate improvements in teamwork and safety culture, which were maintained for two months after training. However, these gains tended to decline at six and twelve months post-intervention, reinforcing the need for regular reinforcement sessions to consolidate a sustained safety culture.

Similarly, although with a shorter training and follow-up period, Stead et al. [75] also found improvements in the culture of safety. The multidisciplinary team initially received an intensive two-and-a-half-day training session and was later responsible for cascading the training to their colleagues. The intervention led to a reduction in the use of mechanical restraints, indicating a direct impact on patient safety. Despite this progress, the study reported difficulties in achieving sustained engagement from hospital management, suggesting that the consolidation of a safety culture requires a strong and continuous organizational commitment.

Kwon & Duzyj [60] implemented the TeamSTEPPS^®^ program over two days, with an evaluation conducted six months later. Although perceptions of teamwork and safety behaviors improved among all participants, differences were noted between professional groups. Nurses provided more critical assessments compared to physicians, indicating a higher level of awareness among the nursing team regarding safety risks. However, no significant differences were observed in clinical outcomes, such as postpartum hemorrhage rates, suggesting that cultural changes may not immediately translate into measurable health outcomes.

Conversely, Aaberg et al. [73] analyzed the implementation of TeamSTEPPS^®^ over a twelve-month period and reported improvements in safety culture, particularly in communication, continuous learning, and incident reporting. The increase in incident reports demonstrated an enhanced reporting culture, indicating a positive shift in transparency and safety accountability following the training.

Thomas & Galla [65] described the large-scale impact of TeamSTEPPS^®^ as part of an organizational strategy. The intervention became a mandatory training program completed over fifteen months. Among the reported outcomes, organizational commitment to safety culture stood out, demonstrating that large-scale integration of the program’s principles depends fundamentally on leadership engagement and institutional support.

Finally, a study conducted in reproductive healthcare settings [55] assessed the implementation and long-term effects of the program over a two-year period. Patients reported higher satisfaction with the care provided, and the study concluded that maintaining TeamSTEPPS^®^-based practices strengthens organizational support and reinforces the sustainability of a safety-oriented culture over time.

In summary, all these studies demonstrate clear benefits of TeamSTEPPS^®^ implementation in reinforcing patient safety culture across different healthcare contexts. However, as highlighted by Hassan et al. [69], the tendency for training effects to diminish over time underscores the importance of continuous education, ongoing team practice, and leadership-driven reinforcement to sustain a robust safety culture.

## 5. Limitations

This review presents several limitations that should be considered when interpreting the reported findings. First, the inclusion of studies conducted in diverse academic and clinical settings and involving different healthcare professional groups introduces heterogeneity that may influence the outcomes reported and limits comparability across studies. The heterogeneity of the studies, including variations in clinical contexts, methodologies, and target populations, limits direct comparisons and the generalizability of results. Additionally, most of the included studies were conducted in settings with different organizational, cultural, and economic realities, further restricting the transferability of findings across contexts.

Another identified limitation is the lack of studies that consistently assess long-term outcomes of the training programs. Finally, the selection of articles restricted to specific languages and databases may have resulted in the exclusion of potentially relevant scientific evidence, and the use of relatively narrow keywords may have limited the scope of the results obtained. In addition, this review may be subject to publication bias, as studies reporting positive outcomes are more likely to be published.

Despite its contributions, this review has limitations that reflect gaps in the current literature, which can be mapped through the PCC framework. Regarding Population, most studies are centered on the doctor-nurse dyad, leaving a gap in evidence concerning non-clinical and administrative staff. In terms of Context, the findings are heavily weighted toward acute care hospitals, with limited data from primary care or long-term care settings. Finally, concerning the Concept, a major limitation is the scarcity of longitudinal data; most evidence focuses on immediate post-training attitudes rather than long-term clinical metrics or sustained behavioral changes. Future research should prioritize these areas to ensure a more holistic and lasting implementation of TeamSTEPPS^®^.

To better capture the long-term impact of TeamSTEPPS^®^, future studies should move beyond short-term attitudes scales. We recommend a longitudinal evaluation framework that combines periodic teamwork climate assessments (e.g., T-TAQ) with objective clinical metrics, such as the frequency of ‘near misses’, fall rates, or the efficiency of clinical handovers (e.g., using SBAR mnemonic). Tracking these metrics over 6 to 12 months would provide a clearer picture of how team training translates into lasting organizational safety culture.

## 6. Conclusions

This scoping review mapped the available evidence regarding the implementation of the TeamSTEPPS^®^ program across academic and clinical settings. Although clear short-term benefits were identified, gaps remain regarding the sustainability of these gains in the medium and long term.

The analysis of the 28 included studies allowed the identification of four thematic categories, which provided a detailed understanding of the importance and outcomes generated by the application of this training method.

The included studies demonstrated that the consolidation of TeamSTEPPS^®^ training as a structured practice depends not only on its implementation across various clinical and non-clinical settings but also on the commitment of organizational leadership.

Strengthening the scientific evidence regarding the effects of TeamSTEPPS^®^ on clinical, organizational, and relational indicators may be further supported through the development of future studies designed to explore these dimensions related to promoting patient safety and improving team-based care delivery.

It was concluded that although long-term training programs proved to be more effective, even short-term interventions can generate immediate improvements in team dynamics and its core components. Several studies emphasized the importance of continuous learning and reinforcement, suggesting avenues for future investigation into how these strategies are maintained over time. Based on the evidence mapped in this review, TeamSTEPPS^®^ should be supported as a continuous change effort, not just as a training course. The studies reviewed suggest that continuous reinforcement, leadership involvement, and monitoring of teamwork and safety routines can help sustain the gains identified.

This scoping review extends previous reviews by integrating evidence from both educational and clinical contexts and by identifying organizational and leadership-related factors that influence the sustainability of TeamSTEPPS^®^ related benefits. These insights may support educators, managers, and policymakers in designing TeamSTEPPS^®^ initiatives as sustained organizational change efforts rather than isolated training interventions.

Across the included studies, the effectiveness of TeamSTEPPS^®^ was primarily demonstrated through a combination of attitudinal, behavioral, organizational, and, less frequently, clinical outcomes. Most studies assessed changes in teamwork attitudes, communication, leadership, and mutual support using validated self-report instruments such as the TeamSTEPPS^®^ Teamwork Attitudes Questionnaire (T-TAQ) and structured observation tools like the Team Performance Observation Tool (TPOT). Behavioral outcomes were commonly evaluated through direct observation of team performance in simulation or real-world settings, focusing on the use of standardized communication tools, briefings, and debriefings. Several studies also examined organizational outcomes, particularly patient safety culture, incident reporting, and organizational learning processes. Fewer studies reported objective clinical outcomes; however, those that did documented improvements in indicators such as response times, adverse event rates, and patient satisfaction. Overall, the findings suggest that while short-term improvements are consistently observed following TeamSTEPPS^®^ training, sustained benefits are more frequently reported in studies incorporating repeated assessments, leadership engagement, and integration of TeamSTEPPS^®^ practices into organizational routines.

## Figures and Tables

**Table 1:** Search strings used in each database.

Databases (Results)	Research Terms
CINHAL Ultimate (138)	Teamstepps AND (benefits OR positive effects OR importance OR impact OR advantages OR outcomes OR MH “Outcome Assessment” OR consequences OR effectiveness OR MH “Teamwork” OR teamwork OR MH “Patient Safety” OR “patient safety” OR MH “Communication” OR communication OR MH “Interprofessional Collaboration” OR “interprofessional collaboration”) AND (Clinical practice settings OR educational contexts)
MEDLINE Ultimate (174)	teamstepps AND [benefits OR positive effects OR importance OR impact OR advantages OR outcomes OR Outcome Assessment OR consequences OR effectiveness OR teamwork OR “Patient Safety” (MeSH Terms) OR “patient safety” OR “Communication” (MeSH Terms) OR communication OR “interprofessional collaboration”] AND (Clinical practice settings OR educational contexts)
SCOPUS (161)	[TITLE-ABS-KEY (teamstepps)] AND [TITLE-ABS-KEY (benefit OR impact OR effectiveness OR consequences OR “positive effects” OR importance OR advantages OR outcomes OR teamwork OR “patient safety” OR communication OR “interprofessional collaboration”)] AND [TITLE-ABS-KEY (“clinical practice” OR education OR educational)]
Psychology and Behavioral Sciences Collection (12)	teamstepps AND (benefits OR “positive effects” OR importance OR impact OR advantages OR outcomes OR consequences OR effectiveness OR teamwork OR communication OR “interprofessional collaboration” OR “patient safety”) AND (education OR educational OR clinical OR practice)
Web of Science (185)	[TS = (teamstepps)] AND [TS = (benefit OR importance OR impact OR outcomes OR consequences OR “positive effects” OR effectiveness OR advantages OR teamwork OR patient safety OR communication OR “interprofessional collaboration”] AND [TS = (“clinical practice” OR education OR educational)]
RCAAP (5)	TeamSTEPPS AND outcomes

**Table 2:** Synthesis of the Contribution of the Included Articles within the Identified Thematic Categories.

Selected Articles	Academic Education, Interprofessional Education, and Simulation	Professional Transition and Professional Development	Clinical Implementation of TeamSTEPPS^®^ in Real-World Settings	Patient Safety Culture as a Central Focus
Aaberg et al. (2019) [72]			**✓**	
Aaberg et al. (2021) [73]			**✓**	**✓**
Ballangrud et al. (2020) [74]			**✓**	
Brock et al. (2013) [52]	**✓**			
Clapper et al. (2018) [53]	**✓**			
Davis et al. (2019) [54]			**✓**	**✓**
Dodge et al. (2021) [55]	**✓**			
Foltz-Ramos et al. (2025) [56]	**✓**			
Forstater et al. (2024) [57]	**✓**			
Fowler et al. (2023) [58]	**✓**			
Gonçalves et al. (2022) [76]		**✓**		
Harvey et al. (2019) [59]		**✓**		**✓**
Hassan et al. (2024) [69]	**✓**			
Jernigan et al. (2016) [51]			**✓**	**✓**
Jitwiriyanont et al. (2025) [70]		**✓**		
Karlsen et al. (2021) [71]			**✓**	
Kwon & Duzyj (2024) [60]	**✓**			
Mahmood et al. (2021) [77]			**✓**	
Matzke et al. (2021) [61]			**✓**	**✓**
Mayer et al. (2011) [62]			**✓**	**✓**
Momim & Nguyen (2023) [63]	**✓**			
Snow et al. (2022) [64]			**✓**	**✓**
Stead et al. (2009) [75]	**✓**			
Thomas & Galla (2013) [65]	**✓**			
Umoren et al. (2017) [66]	**✓**			
Volino et al. (2022) [50]	**✓**			
Weaver et al. (2010) [67]			**✓**	**✓**
Williams et al. (2020) [68]	**✓**			

## Data Availability

The original contributions presented in this study are included in the article/Supplementary Material. Further inquiries can be directed to the corresponding author(s).

## Supplementary Materials

The following supporting information can be downloaded at: https://www.mdpi.com/article/10.3390/nursrep16030079/s1, Supplementary File S1: PRISMA-ScR Checklist; Supplementary File S2: Data extraction table.

## Public Involvement Statement

There was no public involvement in any aspect of this research.

## Guidelines and Standards Statement

This manuscript was drafted against the Preferred Reporting Items for Systematic Reviews and Meta-Analyses Extension for Scoping Reviews (PRISMA-ScR) [49].

## Abbreviations

The following abbreviations are used in this manuscript:

ECMO	Extracorporeal Membrane Oxygenation
ISBAR	Identify, Situation, Background, Assessment and Recommendation
PRISMA-ScR	Preferred Reporting Items for Systematic Reviews and Meta-Analyses Extension for Scoping Reviews
SBAR	Situation Background Assessment Recommendation
SWOT	Strengths, Weaknesses, Opportunities & Threats
T-TAQ	TeamSTEPPS^®^ Teamwork Attitude Questionnaire
TeamSTEPPS^®^	Team, Strategies and Enhance Performance and Patient Safety
TPOT	Team Performance Observation Tool
