# Supplementary material for: Enhancing Teamwork and Patient Safety Through TeamSTEPPS®: A Scoping Review of Benefits in Academic and Clinical Settings
Source: Nurs Rep. 2026 Feb 24;16(3):79. doi: 10.3390/nursrep16030079 (PMC13029451; doi:10.3390/nursrep16030079)
Supplement: Supplementary file 1 [file nursrep-16-00079-s001.zip › Supplementary File S2_data extraction table__8jan com mais artigos.pdf]

Supplementary File S1. data extraction table

	Author(s)<br>Year of publication<br>Country	Title	Objectives	Study Design	Study sample	Context	Relevant concept(s) of the review question<br>Data collection instrument(s)	Main Results
**1**	Aaberg et al.<br>(2019)<br>Norway	A complex teamwork intervention in a surgical ward in Norway.	Explore the impact of an interprofessional teamwork intervention in a surgical ward.	Quasi-experimental study.	Healthcare professionals (physicians, nurses, and nursing assistants).	Intervention and control groups.	The team training was conducted in collaboration with the ward leaders.<br>Instruments: TeamSTEPPS® Teamwork Perceptions Questionnaire (T-TPQ), TeamSTEPPS® Teamwork Attitudes Questionnaire (T-TAQ), Care Decisions in Teams Questionnaire (CSACD-T), Hospital Survey on Patient Safety Culture (HSOPS), and the Quality from the Patient's Perspective (QPP).	Structural changes.
**2**	Aaberg et al.<br>(2021)<br>Norway	A human factors intervention in a hospital evaluating the outcome of a TeamSTEPPS® program in a surgical ward.	Evaluate professional and organizational outcomes.	Quantitative.	Healthcare professionals from a urology and gastrointestinal surgery ward (n = 43).	The team training combined didactic exercises, videos, role-playing, and high-fidelity simulation training.	Mandatory 6-hour interprofessional TeamSTEPPS® training.<br>Instruments: TeamSTEPPS® Teamwork Perceptions Questionnaire (T-TPQ), Collaboration and Satisfaction about Care Decisions in Teams (CSACD-T), and the Hospital Survey on Patient Safety Culture (HSOPS).	After 12 months of intervention, significant improvements were observed in three teamwork dimensions: Situation Monitoring, Mutual Support, and Communication.<br>No significant changes were found in the professional outcome Team Decision-Making (CSACD-T).<br>Patient safety culture results (HSOPS) showed significantly improved scores in two dimensions: Organizational Learning and Continuous Improvement and Communication Openness. During the study period, the number of reported adverse events increased, indicating an improvement in the reporting culture.
**3**	Ballangrund et al.<br>(2020)<br>Norway	Longitudinal team training programme in a Norwegian surgical ward: a qualitative study of nurses' and physicians' experiences with teamwork skills.	Describe professional experiences following TeamSTEPPS® training.	Qualitative.	Healthcare professionals (physicians, nurses, and nursing assistants).	Surgical unit (gastrointestinal and urology surgery).<br>The training program was implemented in three phases: assessment, planning, training, and implementation.	Four professionals completed the Master Training in TeamSTEPPS®.<br>Instrument: Interview.	Improved interprofessional information exchange.<br>Enhanced leadership.<br>Effective resource management.<br>Standardized communication.
**4**	Clapper et al.<br>(2018)<br>USA	A Saturated Approach to the Four-Phase, Brain-Based Simulation Framework for TeamSTEPPS® in a Pediatric Medicine Unit.	Evaluate the implementation of the TeamSTEPPS® program in a pediatric unit.	Quantitative.	Nurses, physicians, pharmacists, respiratory therapists, and unit staff (n = 547).	Implementation of the TeamSTEPPS® program in a pediatric medicine unit.	The training lasted 8 weeks.<br>Instruments: A researcher-developed test to assess knowledge about TeamSTEPPS® and the TeamSTEPPS® Team Performance Observation Tool (T-TPO).	Knowledge about TeamSTEPPS and team performance improved after the intervention.<br>Mutual support increased.
**5**	Davis et al.<br>(2019)<br>USA	The Integration of TeamSTEPPS® Into Interprofessional Education Curricula at 3 Academic Health Centers.	Describe how three universities integrated the TeamSTEPPS® program into their interprofessional education curricula.	Qualitative.	Students from different health disciplines.	The three institutions implemented different training models.	Instruments: TeamSTEPPS® Team Performance Observation Tool (T-TPO), TeamSTEPPS® Teamwork Attitudes Questionnaire, and a questionnaire on satisfaction and competencies for collaborative practice.	The T-TPO categories showed a significant improvement in performance.
**6**	Dodge et al.<br>(2020)<br>USA	Long-term effects of teamwork training on communication and teamwork climate in ambulatory reproductive health care.	Describe team communication and the perceptions of both staff and patients regarding teamwork, two years after the implementation of TeamSTEPPS®.	Quantitative.	Reproductive health centers that implemented the TeamSTEPPS® program.	The preparatory activities included forming a change team, participating in informational webinars, and conducting a SWOT analysis.	Instruments: TeamSTEPPS® Teamwork Perceptions Questionnaire (T-TPQ), Patients' Insights and Views of Teamwork (PIVOT), and the analysis of demographic characteristics and satisfaction levels.	Standardized communication.<br>Increased patient satisfaction.<br>Team meetings.
**7**	Foltz-Ramos et al.<br>(2025)<br>USA	Preparing future healthcare professionals: Role-play and simulation in TeamSTEPPS® training.	The study aimed to evaluate the effectiveness of incorporating a role-play session before clinical simulation within an interprofessional TeamSTEPPS® educational program.	Quasi-experimental study.	466 students from seven health professions programs.	Conducted at a large public university in the United States as part of an interprofessional education program integrating TeamSTEPPS® training.	Interprofessional education, teamwork, communication, role-play, simulation, TeamSTEPPS® training.<br>Instruments: TeamSTEPPS® Teamwork Attitudes Questionnaire (T-TAQ), Retrospective pre/post confidence survey on TeamSTEPPS® tools, and 21-item post-program evaluation survey developed by the research team.	Students who participated in role-play before simulation showed higher confidence in using TeamSTEPPS® tools, while overall teamwork attitudes and satisfaction with the interprofessional program improved across all participants.
8	Forstater et al.<br>(2024)<br>USA	A TeamSTEPPS®-Based Simulation Delivered Virtually Provides Teamwork and Communication Outcomes Comparable to In-Person Instruction	To evaluate whether a virtually delivered TeamSTEPPS®-based interprofessional simulation produces teamwork and communication outcomes comparable to those achieved through in-person instruction.	Quasi-experimental.	1,158 pre-clinical students.	Interprofessional education setting.	Interprofessional education, teamwork, communication, patient safety, and virtual simulation training.<br>Instruments: Pre- and post-simulation surveys assessing teamwork knowledge and self-perceived teamwork ability, Jefferson Team Observation Guide (JTOG®) for team performance evaluation, and Qualitative feedback questionnaire on roles, responsibilities, and satisfaction with the simulation.	Virtual TeamSTEPPS®-based simulation significantly improved students' teamwork knowledge, confidence, and communication skills, achieving outcomes comparable to in-person training.
**9**	Fowler et al.<br>(2023)<br>USA	Alignment of an interprofessional student learning experience with a hospital quality improvement initiative.	Highlight an innovative training program in interprofessional education.	Quasi-experimental.	Students from different health disciplines.	The TeamSTEPPS® training was integrated into a mandatory interprofessional project.	Instruments: TeamSTEPPS® Team Performance Observation Tool (T-TPO).	Significant improvements in teamwork performance.<br>These improvements continued after the intervention and were sustained after 15 months.<br>Increased use of teamwork skills.<br>Enhanced understanding of the importance of interprofessional collaboration.
**10**	Gonçalves et al.<br>(2022)<br>Brazil	Teamwork in Pediatric Resuscitation: Training Medical Students on High Fidelity Simulation.	Evaluate the contribution of TeamSTEPPS® to technical and non-technical performance.	Quasi-experimental.	Medical students.	Control and intervention groups.<br>Both groups received CPR training, but the intervention group also received TeamSTEPPS® training.	Instruments: Theoretical pre-test, theoretical and practical post-tests, training satisfaction survey, checklist, and video analysis.	In the practical test, the intervention group achieved higher scores.<br>Teamwork training improved the intervention group's technical performance.
**11**	Harvey et al.<br>(2019)<br>USA	Impact of Advanced Nurse Teamwork Training on Trauma Team Performance.	Determine the impact of TeamSTEPPS® in a trauma nursing course.	Quasi-experimental.	Nurses.	Use of simulation as a training method.	Instruments: Trauma and TeamSTEPPS® Knowledge Test, Trauma Nurse Core Course (TNCC), Trauma Nurse Process (TNP) Skill Assessment, Trauma RN Confidence Survey, Brief TeamSTEPPS® Teamwork Perceptions Questionnaire (Brief T-TPQ), Trauma Performance Observation Tool (TPOT), and Trauma Registry Data.	Positive effect on nurses' confidence.<br>Performance declined 6 to 12 months after the initial training.
**12**	Hassan et al.<br>(2024)<br>Egypt	Evaluating the Effect of TeamSTEPPS® on Teamwork Perceptions and Patient Safety Culture among Newly Graduated Nurses.	Evaluate the influence of TeamSTEPPS® on teamwork perceptions and patient safety culture among newly graduated nurses.	Quasi-experimental.	132 newly graduated nurses.	Conducted in a university hospital.	The TeamSTEPPS® training program was delivered over seven weeks.<br>Instruments: TeamSTEPPS® Teamwork Perceptions Questionnaire (T-TPQ), and the Hospital Survey on Patient Safety Culture (HSPSC).	Significant improvement in mean scores for teamwork perceptions and patient safety culture.
**13**	Jitwiriyanont<br>et al.<br>(2025)<br>Thailand	Advancing Politeness and Assertive Communication Through Tone of Voice in Crisis Team Situations: Pre-Post Acoustic Analysis Study of Team and Strategies to Enhance Performance and Patient Safety (TeamSTEPPS®) Virtual Simulation for Interprofessional Education in Health Care Undergraduate Students.	To assess the impact of TeamSTEPPS® virtual simulation on polite and assertive communication among healthcare students.	Quasi-experimental.	29 undergraduate clinical students.	The study took place in a simulated emergency department scenario (academic context).	Interprofessional communication, teamwork, politeness, assertiveness, tone of voice, and virtual simulation (TeamSTEPPS®).<br>Instrument: Audio recordings of pre- and post-training sessions analyzed with Praat software to extract 11 acoustic parameters (pitch/F0, intensity, and duration) for acoustic voice analysis.	TeamSTEPPS® virtual simulation improved students' tone of voice, enhancing politeness, assertiveness, and interprofessional communication.
**14**	Stead et al.<br>(2009)<br>Australia	Teams communicating through STEPPS.	Evaluate the implementation of TeamSTEPPS® in a mental health unit.	Qualitative.	Multidisciplinary team.	Intensive 2.5-day training.	Instruments: Observation, Hospital Survey on Patient Safety Culture (HSPSC), and a questionnaire based on the Total Team Assessment Questionnaire.	Restructuring of multidisciplinary meetings.<br>Introduction of standardized communication tools.<br>Reduction in restraint rates.
**15**	Kwon & Duzyj<br>(2024)<br>USA	The Impact of TeamSTEPPS® Training on Obstetric Team Attitudes and Outcomes on the Labor and Delivery Unit of a Regional Perinatal Center.	Investigate the outcomes of TeamSTEPPS® on teamwork perceptions and patient safety results in obstetrics.	Quantitative.	Physicians and nurses.	Physicians and nurses received TeamSTEPPS® training adapted to the local context.<br>Educational session of 180 minutes divided over two days.	Instruments: TeamSTEPPS Teamwork Perceptions Questionnaire and quantitative data review.	Nurses reported a more negative perception of safety behaviors.<br>No significant differences in hemorrhage outcomes were observed compared to the pre-training period.

	Author(s) Year of publication Country	Title	Objectives	Study Design	Study sample	Context	Relevant concept(s) of the review question Data collection instrument(s)	Main Results
16	Matzke et al. (2021) USA	Incorporating TeamSTEPPS® training to improve staff collaboration in an academic level I emergency and trauma center.	Evaluate the effect of TeamSTEPPS® in an urgent care center.	Quantitative.	Nurses and healthcare assistants (n = 34).	Project implemented in an emergency department. The TeamSTEPPS® curriculum was adapted to the emergency care context to develop a 60-minute educational session.	Innovative project with a 1-hour training session.<br>Instrument: TeamSTEPPS® Teamwork Perceptions Questionnaire (T-TPQ).	Significant improvement in teamwork and communication.<br>No significant change was found for leadership, team structure, or mutual support.<br>A 60-minute session proved effective in improving professionals' short-term perceptions.
17	Mayer et al. (2011) USA	Evaluating Efforts to Optimize TeamSTEPPS® Implementation in Surgical and Pediatric Intensive Care Units.	Implement and evaluate TeamSTEPPS® in a pediatric intensive care unit.	Quantitative.	Physicians, nurses, and physiotherapists.	2.5-hour training session.	Although the basic TeamSTEPPS curriculum is designed for 4 to 6 hours, the program developers acknowledge that little is known about the most effective approach for teaching the material.<br>Instruments: Interviews, Teamwork Evaluation of Non-Technical Skills (TENTS) observation tool, Hospital Survey on Patient Safety Culture (HSPSC), Employee Opinion Survey (EOS), and the National Database of Nursing Quality Indicators (NDNQI).	Improved teamwork experience.<br>Better communication and performance.<br>The average time to initiate patients on ECMO decreased.<br>Hospital infection rates decreased.
18	Momim & Nguyen (2023) USA	Assessing the Efficacy of Teamstepps® and Simulation in Interprofessional Education.	Evaluate the effectiveness of TeamSTEPPS® training and simulation in improving interprofessional competencies among medical and respiratory therapy students.	Quantitative.	Medical and respiratory therapy students.	Interprofessional education in an academic setting, with an emphasis on clinical simulation.	Pre- and post-intervention tests.<br>Observational assessment of interprofessional skills.<br>Instrument: Performance Assessment for Communication and Teamwork (PACT).	Significant improvements in interprofessional skills among medical and respiratory therapy students.<br>The TeamSTEPPS® didactic sessions, simulation, and debriefing were effective for both groups of students.
19	Snow et al. (2022) USA	Implementation of a virtual simulation-based teamwork training program for emergency events in the perioperative setting.	Implement communication standardization through TeamSTEPPS®.<br>Reflect on effective communication.<br>Apply communication skills.	Quantitative.	Interprofessional team in the perioperative setting.	Need to improve team communication.	Experiential learning using pre-recorded simulations. No reference to the assessment instrument used.	Communication among professionals improved.
20	Thomas & Galla (2013) USA	Building a culture of safety through team training and engagement.	Build a safety culture and emphasize the relevance of TeamSTEPPS® as essential within an organizational context.	Quantitative.	Members of the interprofessional team.	A corporate team with expertise in research and Evidence-Based Practice was trained in TeamSTEPPS®.	15-month training program.<br>TeamSTEPPS® is included in the mandatory annual training and orientation program.<br>Instrument: Hospital Survey on Patient Safety Culture (HSOPSC).	Improvements in feedback, communication, general perceptions, and safety.<br>It is essential for managers to advocate for the program to ensure its successful dissemination.<br>Physicians responded more positively to training delivered by other physicians.
21	Umoren et al. (2017) USA	TeamSTEPPS® Virtual Teams: Interactive Virtual Team Training and Practice for Health Professional Learners.	Prepare students for the delivery of safe and effective care.	Qualitative.	Nursing and medical students.	To enhance the interprofessional education curriculum, faculty from various institutions and specialties developed a series of eight cases.	Instrument: TeamSTEPPS® Virtual Teams — a virtual simulation platform that enables multi-player interaction as well as individual participation in a series of pre-programmed interactions with a virtual healthcare team.	Standardized communication.<br>Knowledge dissemination.<br>Innovative teaching approach.
22	Weaver et al. (2010) USA	Does Teamwork Improve Performance in the Operating Room? A Multilevel Evaluation.	Optimize teamwork behaviour and evaluate the impact of TeamSTEPPS® on teamwork training.	Quasi-experimental.	Operating room teams.	Intervention and control groups.<br>The training included a 4-hour didactic session.	Instruments: Observations, Medical Performance Assessment Tool for Communication and Teamwork (MedPACT), Hospital Survey on Patient Safety Culture (HSOPS), and Operating Room Management Attitudes Questionnaire (ORMAQ).	Only 52% felt confident enough to teach the methodology.<br>Perceptions of teamwork among the trained group increased.<br>The hospital integrated TeamSTEPPS® into the training of all staff members.
23	Williams et al. (2020) USA	Preparing students for clinical practice: The impact of a TeamSTEPPS® inter professional education session.	Evaluate the impact of interprofessional education on students' perceptions.	Quantitative.	Medical and nursing students.	Interprofessional education is an integral part of the medical and nursing curricula throughout all academic years.<br>One week of patient safety training.<br>TeamSTEPPS® training using simulation scenarios and standardized communication.	Instrument: Readiness for Interprofessional Learning Scale (RIPLS).	Greater willingness and perceived need to share knowledge and skills with other students.<br>Improvements in reflective capacity.<br>The results indicate a deficit in the definition and clarity of professional roles.
24	Volino et al. (2022) USA	Evaluation of Interprofessional Education on Effective Communication Between Pharmacy and Physician Assistant Students.	Evaluate he effect of implementing the TeamSTEPPS® program on students' self-perceived skills and their perceptions of interprofessional communication and teamwork.	Quantitative.	Medical and pharmacy students.	Educational/academic context, as part of an interprofessional education activity (IPE) integrated into the university curriculum, with simulation and peer feedback.	Instrument: Performance Assessment for Communication and Teamwork (PACT)	Statistically significant improvements ($p < 0.004$) were observed in most assessed domains after the TeamSTEPPS® intervention.<br>Greater gains were seen in the Attitudes and Perceived Skills domains.<br>The Mutual Support domain did not show a significant overall change.<br>The application of TeamSTEPPS®, combined with simulation and peer feedback, improves students' perception of effective communication and teamwork.
25	Karlsen et al. 2021 Norway	Bachelor of nursing students' attitudes toward teamwork in healthcare: The impact of implementing a TeamSTEPPS® team training program — A longitudinal, quasi-experimental study	Explore the impact of implementing the TeamSTEPPS® program on the attitudes of nursing students.	Quasi-experimental.	Nursing Students.	Educational/academic context, integrated into an undergraduate Nursing course, including theoretical activities, simulation, skills training, and clinical placements.	The Teamwork Attitudes Questionnaire (T-TAQ) was applied before the intervention (T0), after ten months (T1), and after 24 months (T2).<br>Instrument: TeamSTEPPS® Teamwork Attitudes Questionnaire (T-TAQ)	**The intervention group showed statistically significant improvements in attitudes toward teamwork at both 10 and 24 months.**<br>**The T-TAQ score increased significantly in the intervention group.**<br>**The greatest positive effects were observed in the Team Structure, Leadership, and Situation Monitoring dimensions.**<br>**The results suggest that the continuous and longitudinal integration of TeamSTEPPS® into the curriculum promotes sustainable changes in students' attitudes.**
26	Mahmood et al. 2021 India	Interprofessional simulation education to enhance teamwork and communication skills among medical and nursing undergraduates using the TeamSTEPPS® framework	Evaluate an interprofessional simulation education module for undergraduate medical and nursing students, focusing on teamwork and communication skills.	Quasi-experimental.	Medical and nursing students.	Academic context, developed in a clinical simulation center, integrated into undergraduate health training.	Instrument: TeamSTEPPS® 2.0 Performance Observation Tool (TPOT); UW Health Sciences Performance Assessment Communication and Teamwork Tools Set; Interprofessional Education Collaborative (IPEC) Competency Self-Assessment Tool and Interprofessional Socialization and Valuing Scale (ISVS).	**Statistically significant improvements ($p < 0.001$) in team performance across all domains of TeamSTEPPS®.**<br>**Significant increase in interprofessional skills and collaborative attitudes.**<br>**The importance of interprofessional simulation, the positive impact of structured communication tools (ISBAR) on patient safety, and greater awareness of professional roles were highlighted.**<br>**Sustained positive evaluation two weeks after the intervention.**
27	Jernigan et al. 2016 USA	Student Outcomes Associated with an Interprofessional Program Incorporating TeamSTEPPS®	Develop and evaluate an innovative pilot program for interprofessional education incorporating TeamSTEPPS® for healthcare students.	Quasi-experimental.	Students from the health field: Medicine, Nursing, Pharmacy, Social Work, Physiotherapy, Occupational Therapy, Speech Therapy, Dietetics, Biomedical Sciences, among others.	Academic context, with institutional implementation of a mandatory foundational interprofessional education program, prior to autonomous clinical practice.	Instrument: TeamSTEPPS® Teamwork Attitudes Questionnaire (TAQ), Test on knowledge retention regarding TeamSTEPPS and Satisfaction questionnaire.	**Statistically significant improvements in attitudes toward teamwork.**<br>**All five domains of TeamSTEPPS® (team structure, leadership, situation monitoring, mutual support, and communication) showed significant improvements at Level 1.**<br>**Over 80% of students demonstrated acquisition of specific knowledge in TeamSTEPPS®.**<br>**Participants reported greater appreciation for interprofessional collaboration and an intention to apply TeamSTEPPS® tools (SBAR, CUS, briefs, huddles, and check-backs) in future practice.**<br>**The program proved viable, well-accepted, and scalable in an institutional academic context.**
28	Brock et al. 2013 USA	Interprofessional education in team communication: working together to improve patient safety	**Describe and demonstrate the effectiveness of an innovative interprofessional training effort using simulation.**	Quasi-experimental	Medicine, nursing and pharmacy students.	Academic educational context, integrated into an interprofessional activity, carried out in clinical simulation environments.	Instrument: Attitudes, Motivation, Utility and Self-Efficacy (AMUSE) and TeamSTEPPS Teamwork Attitudes Questionnaire (T-TAQ)	**Statistically significant improvement in: Attitudes towards teamwork, motivation for interprofessional communication, perception of the usefulness of training and a significant increase in knowledge about: Team structure, monitoring a situation, mutual support and communication.**<br>**Interprofessional training based on TeamSTEPPS® has developed consistent positive effects on students' attitudes and knowledge.**
